# Prof Mladen Petrovečki, MD, PhD, 1960-2016

**DOI:** 10.3325/cmj.2016.57.522

**Published:** 2016-10

**Figure 1:**
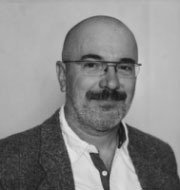
Prof Mladen Petrovečki, MD, PhD, 1960-2016

Rare are the ones who succeed concurrently in more than one professional field; they are as rare as those who are equally passionate about their career, hobbies, family, and friends. Professor Mladen Petrovečki (Figure 1) was both. He was tireless in sharing his knowledge and ideas, which at times led to intriguing discussions on statistics, medicine, patient care, philosophy of science, and the Croatian language. In his private life he was passionate about his grandchildren, trains, Star Trek, and LEGOs. Above all, he had a sublime sense of humor and was able to turn serious work into play. His life was cut horribly short at the age of 57, leaving his wife Vedrana, son Marko, and two grandchildren, Filip and Laura.

Mladen Petrovečki was Full Professor of Medical Informatics at the Department of Medical Informatics, Rijeka University Faculty of Medicine in Rijeka, and Head of the Immunology Division at the Department of Laboratory Diagnostics, Dubrava Hospital in Zagreb. He was experienced in medical informatics education, computer-assisted database formation, biomedical statistical data analysis, and teaching of research integrity. He was the first Statistical and Research Integrity Editor in the *Croatian Medical Journal* and statistical advisor in the *Lancet* and *Acta Stomatologica Croatica*. In the 2004-2006 he was Assistant Minister of Science, Education and Sports of the Republic of Croatia. He published many scientific publications and wrote a book of short stories in Croatian “Zloduh.” During Prof. Petrovečki’s decades-long work as a statistical editor in the *Croatian Medical Journal*, we relied greatly on his judgment and advice. His contribution to both education of younger colleagues and reviewing articles for methodological issues was indispensable – all this increased the Journal’s quality and impact. Prof. Petrovečki was one of those extraordinary people who changed their surroundings for the better, and for this achievement he should be remembered and thanked.

